# Exploring the Ecological Preferences and Essential Oil Variability in Wild-Growing Populations of the Endangered Local Greek Endemic *Thymus holosericeus* (Lamiaceae)

**DOI:** 10.3390/plants12020348

**Published:** 2023-01-11

**Authors:** Olga S. Tsiftsoglou, Rafaela Stagiopoulou, Nikos Krigas, Diamanto Lazari

**Affiliations:** 1 Laboratory of Pharmacognosy, School of Pharmacy, Aristotle University of Thessaloniki, 54124 Thessaloniki, Greece; 2 Institute of Plant Breeding and Genetic Resources, Hellenic Agricultural Organization DEMETER-Thermi, 57001 Thessaloniki, Greece

**Keywords:** TLC analysis, NMR analysis, linalool, carvacrol, geraniol, thymol, *p*-cymene, chemotypes, chemodiversity, ecological profile, climatic correlations, canonical correlation analysis

## Abstract

*Thymus holosericeus* Čelak. (Lamiaceae) is a taxonomically isolated and endangered local endemic thyme species which is geographically isolated in four Ionian Islands (West Greece). The present study investigated the essential oil (EO) composition, the ecological preferences, and their correlations in three *T. holosericeus* wild-growing populations from Zakynthos (ΤH-Z), Cephalonia (ΤH-C) and Lefkada (ΤH-L). The variations in essential oil yield and the composition of *T. holosericeus* populations were evaluated using hydrodistillation, GC/MS, TLC and NMR analysis. The climatic conditions of each sample were organized and analyzed in RStudio with the raster package and in SPSS with Pearson’s Canonical Correlation Analysis (CCA), respectively. The aerial parts of the plants varied in EO yields from 1.92 to 2.28% *w*/*v*. The analysis of EO constituents revealed noteworthy qualitative and quantitative inter-population variation. The composition of EOs revealed the presence of linalool (82.77%) and borneol (5.95%) as major compounds in ΤH-Z, while carvacrol (35.34%), geraniol (23.98%), linalool (14.37%), borneol (5.66%), thymol (4.27%) and *p*-cymene (4.08%) were the main compounds in ΤH-C and linalool (40.37%), geraniol (39.42%) and borneol (5.20%) were dominant components in ΤH-L. The results of the gas chromatography procedure have also been confirmed by ^1^H and ^13^C-NMR and TLC analysis. The ecological profile showed an average annual precipitation of 942 ± 18.33 mm and the temperature limits in which *T. holosericeus* seems to adapt to are 6.80± 1.08 °C 27.70 ± 0.70 °C. Regarding the examined samples, TH-C was adapted to the driest summer and coldest winter conditions, TH-Z was adapted to the lowest annual precipitation with the most complex climatic conditions, and TH-L was adapted to the highest summer temperatures with the lowest precipitation in the wettest period of the year. For each sampled population, the CCA identified the association of the samples’ EOs composition with the prevailing local environmental conditions.

## 1. Introduction

Among the aromatic plants belonging to the Lamiaceae family, *Thymus* L. is one of most important genera in terms of species richness and widespread uses in everyday life or at an industrial scale. Both extracts and essential oils isolated from either wild-growing or cultivated *Thymus* species are or can potentially be used in cosmetics or in the pharmaceutical and food industries as natural preservatives due to their biological activities [1]. Species of the genus *Thymus* worldwide are usually grouped in eight different sections (sectiones in Latin) and are distributed across Eurasia, North and East Africa, and southern Greenland, but the great majority of the taxa (species and subspecies) is centered around the Mediterranean region [1].

While many species are to be found in the Mediterranean region [2], members of only half of the sectiones are present in Greece (Sect. *Teucrioides*, *Pseudothymbra*, *Hyphodromi* and *Serpyllum*). Several studies in Greece have investigated the volatile constituents of different *Thymus* members, especially in Sectio *Serpyllum* (e.g., [3]) and Sectio *Hyphodromi*. Among members of the latter, the wild-growing populations of *T. atticus* Čelak. present considerable variation in volatile constituents and its essential oils (EOs) are poor in thymol/carvacrol and rich in sesquiterpenes such as (E)-nerolidol, germacrene D, (E)-caryophyllene and caryophyllene oxide but rich in monoterpenes such as 1,8-cineole, *α*-pinene and camphene [4]. The closely related *T. parnassicus* Halácsy shows remarkable qualitative stability in the volatile constituents of the wild-growing populations, including a mixed chemotype with thymol/*p*-cymene/carvacrol and (E)-caryophyllene as a prominent compound (8.5–13.1%) [4]. *T. samius* Ronniger & Rech. f. is characterized by the presence of germacrene D (26.4%) and *β*-bisabolene (22.7%), which constitute almost half its ΕO [4]. *T. zygioides* Griseb. var. *lycaonicus* (Čelak.) Ronniger is characterized by the abundance of *p*-cymene (19.4%), thymol (19.5%) and *γ*-terpinene (17.2%), with *p*-cymene and *γ*-terpinene being the precursors of the phenol thymol in the biochemical pathway [5].

Although most of the Greek *Thymus* taxa belong to Sect. *Serpyllum* (13 species and subspecies), only four rare and range-restricted taxa belong to Sect. *Teucrioides*. Among the latter, two distinct members of Subsectionem *Thymbropsis* are to be found in Greece, namely, *Thymus laconicus* Jalas (endemic in parts of south Peloponnese) and *Thymus holosericeus* Čelak., which is confined to some of the Ionian Islands (Western Greece). *T. holosericeus* (Lamiaceae, Sect. *Teucrioides,* Subsect. *Thymbropsis*) is a local endemic and taxonomically isolated native thyme species of Greece forming geographically isolated wild-growing populations in Cephalonia, Zakynthos (Zante), Lefkada and Atokos islands of the Ionian Archipelago (https://floraionica.univie.ac.at/index.php?site=2&taxa_id=8154&#tt30, accessed on 13 July 2022). Therefore, current extinction risk assessments classify this local thyme species of Greece as “endangered” [6].

Previous studies regarding *T. holosericeus* have focused either on chromosome numbers (2*n* = 28; [7]), species-specific propagation protocols for ex situ conservation with the use GIS (Geographical Information Systems) [8] or some agronomical traits and basic phytochemical analysis of selected compounds [8,9], thus representing preliminary attempts of its cultivation and evaluation in man-made settings. Despite the research efforts made to date, detailed profiling regarding the EOs of *T. holosericeus* is still in need due to the absence of information regarding its natural EO variability.

In this framework, the first aim of the current investigation was to provide a detailed and validated profile of the essential oils of *T. holosericeus* from different insular areas of the Ionian Sea. The second aim was to develop a detailed and organized profile unveiling the ecological preferences of *T. holosericeus* as well as detecting possible correlations of its essential oil composition and respective bioclimatic data.

## 2. Results and Discussion

It is known that the essential oils of plant species directly correspond to their local environment, reflecting plant responses to drought, intense radiation, high temperature, and soil composition, among others [10]. Additionally, the qualitative characteristics of the essential oils are closely related to environmental factors which result in different plant individuals or populations of the same species having a special composition of essential oils due to their location in which they thrive and their adaptability to local environmental stress factors [10,11]. Consequently, detailed knowledge about essential oil variability and the associated climatic conditions required for the wild-growing occurrence of a given species in a specific place are mandatory, both for developing species-specific conservation actions and sustainable exploitation strategies [12,13].

The study herein has furnished new data for future chemotaxonomical studies in the genus Thymus with the aim of facilitating the sustainable exploitation of T. holosericeus as a new and local endemic aromatic-medicinal plant with interesting potential in various economic sectors [14,15,16].

### 2.1. Composition of the Studied Essential Oils

This study reports a detailed profile of the EOs of *T. holosericeus* for the first time. The extracted oil was yellowish in color and had an aromatic odor. The yields of the volatile fractions obtained from *T. holosericeus* aerial parts were 1.93% (TH-Z), 2.28% (TH-C) and 1.92% (TH-L) (*v*/*w*), respectively, on a dry weight basis (Table 1). These yields are higher than those reported [8,9] for cultivated *T. holosericeus* in Attica and somewhat lower than that of some wild-growing populations in Cephalonia (3.80%) [8].

The different components (*n* = 40) of the essential oils of the studied samples of wild-growing *T. holosericeus* from the Ionian islands, Greece are listed in Table 1 according to the increasing retention times, comprising >99% of the total. The more complex essential oils were those obtained in plant material from Cephalonia (TH-C) with 32 compounds, followed by TH-L and TH-Z with 30 and 28 compounds, respectively. In contrast to previously studied EOs from wild-growing Greek *Thymus* species [4,5], the samples examined herein from *T. holosericeus* seem to be less complex with rather lower amounts of sesquiterpenes. Chemical variation among the studied populations of *T. holosericeus* was also high. Monoterpene oxygenated compounds were found in a considerable amount in every examined EO of *T. holosericeus*. The highest ratio was found in the EO obtained from the plants collected from Zakynthos (92.08%) and the lowest from plants from Cephalonia (40.40%), with linalool being among the dominant compounds in this class (14.37–82.77%). The percentage of sesquiterpene hydrocarbons as well as oxygenated sesquiterpenes was low in every examination. The percentage of monoterpenes (6.85%) and aromatic (39.61%) compounds was higher in material collected from Cephalonia (TH-C) compared to the other two samples. The results of the present study showed qualitative and quantitative differences between the EOs of *T. holosericeus*, thus indicating that environmental factors may strongly influence its chemical composition. Only 16 out of 40 compounds (40%) were common the three population samples, but in different proportions.

Some constituents of the three studied Eos, such as borneol, were found at similar amounts (from 5.20% in TH-L to 5.95% in TH-Z), while the same was true for other minor constituents of the Eos, such as *α*-terpineol, *α*-pinene, camphene, myrcene and others (Table 1). Interestingly, many qualitative differences were detected among the studied EOs of *T. holosericeus* from three different islands. Linalool (Table 1) was almost 6-fold in TH-Z (82.77%) and almost 3-fold in TH-L (40.37%) compared to TH-C (14.37%). A high content of geraniol was detected in TH-C (23.98%) and TH-L (39.42%) compared to its absence in TH-Z. A high content of carvacrol was found in TH-C (35.34%) compared to TH-Z (1.07%) and TH-L (0.28%), which is comparatively higher than that previously reported in cultivated material grown in Attica (20.26%) but lower in respect to wild-growing material from Cephalonia (86.62%) [8,9]. Furthermore, a relatively high content of thymol was evidenced in TH-C (4.27%) compared to TH-Z (0.31%) and TH-L (0.45%), being actually four-fold higher compared to previous studies referring to cultivated material in Attica [9]. A noteworthy proportion of *p*-cymene (4.08%) was detected in TH-C and quite smaller quantities were found in TH-Z (0.32%), while it was not detected in TH-L (Table 1); these were similar or higher values with regard to the cultivated material in Attica (0.28–0.32%) or wild-growing material from Cephalonia (2.28% in [8,9]). A higher amount of *γ*-terpinene (1.05%) was found in TH-C compared to TH-Z (0.32%) and TH-L (0.6%), while rather higher amounts have been reported in cultivated material from Attica (0.48%) or wild-growing material from Cephalonia (1.58%) [8,9]. Camphor was pronounced in TH-L (2.03%) compared to very limited amounts in TH-Z (0.10%) and mere traces found in TH-C. Moreover, *β*-Caryophyllene was higher in TH-Z (2.87%) and TH-L (2.36%) compared to TH-C (1.83%), all of which are higher in respect to cultivated material in Attica (0.74%) or wild-growing populations (0.31%) from Cephalonia [8,9]. Low amounts of terpinen-4-ol (0.69%) were detected in TH-L, intermediate levels (1.61%) in TH-Z and higher (2.43%) in TH-C (Table 1). Such qualitative differences are reported for the first-time. With regard to previously reported studies [8,9], the composition of EO volatiles in both wild-growing and cultivated *T. holosericeus* from Cephalonia show several differences. For instance, carvacrol and geraniol were found to be the major constituents of the EO in material from Cephalonia, both in the present research and in previous studies; however, the relative amounts of these two corresponding compounds differed greatly. Moreover, the chemotype of EOs extracted from wild-growing material from Cephalonia and cultivated material in Spata (Attica) is pure [8,9] in contrast to the findings of the present study.

Greek members of Subsect. *Thymbropsis* have not been studied to date. However, other members of this group of thyme plants are found in other Balkan countries or in Turkey and may serve as a basis for comparisons. For example, the EOs of *T. syriacus* Boiss. [17,18,19,20] are rich in aromatic monoterpenes (carvacrol and thymol) while those of *T. cilicicus* Boiss. & Balansa are characterized by the presence of monoterpene compounds and mixed chemotype [17,18,21]. Among other members of Subsect. *Thymbropsis*, the EOs extracted from *T. eigii* (Zohary & P.H. Davis) Jalas [22,23], *T. cariensis* Hub.-Mor. & Jalas [24] as well as from varieties of *T. sipyleus* Boiss. subsp. *sipyleus* such as var. *rosulans* (Borbás) Jalas [25], var. *davisianus* Ronniger [26], var. *sipyleus* [22,25,27] and varieties of *T. leucostomus* Hausskn. & Velen. such as var. *gypsaceus* Jalas [28], var. *leucospomus* [29] and var. *argillaceus* Jalas [24], all seem to be mainly characterized by the presence of monoterpene compounds showing remarkable chemical polymorphism in volatile constituents. All these geographically distant taxa are taxonomically close relatives of *T. holosericeus* within the Subsect. *Thymbropsis* [2].

To date, both pure and mixed chemotypes have been reported in members of the genus *Thymus,* indicating the existence of “pure chemotypes” when a major constituent is >50% of the total EO composition and “mixed chemotypes” with EO consisting of two or more dominant compounds, each <50% of the volatile oil [30]. In such terms, the sample ΤH-Ζ of the present study was characterized by high percentages of linalool (82.77%) and thus was classified as belonging to the linalool chemotype. The other two samples were both defined as mixed chemotype. The sample TH-C showed high carvacrol (35.34%) and geraniol (23.98%) content and thus was classified to the carvacrol/geraniol chemotype. The sample TH-L was classified to the linalool/geraniol chemotype (40.37/39.42%, respectively).

### 2.2. Nuclear Magnetic Resonance Analysis of the Studied Essential Oils

**Figure 1:**
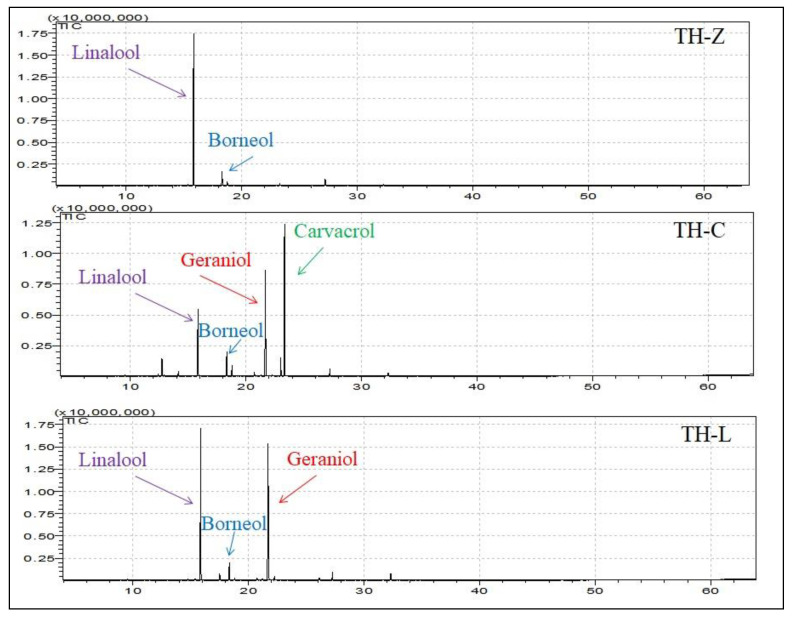
GC-MS chromatographs from the analysis of the essential oils of *Thymus holosericeus* collected from three Ionian Islands, Greece (TH-Z: Zakynthos; TH-C: Cephalonia, TH-L: Lefkada) during full-flowering (July 2018).

**Figure 2:**
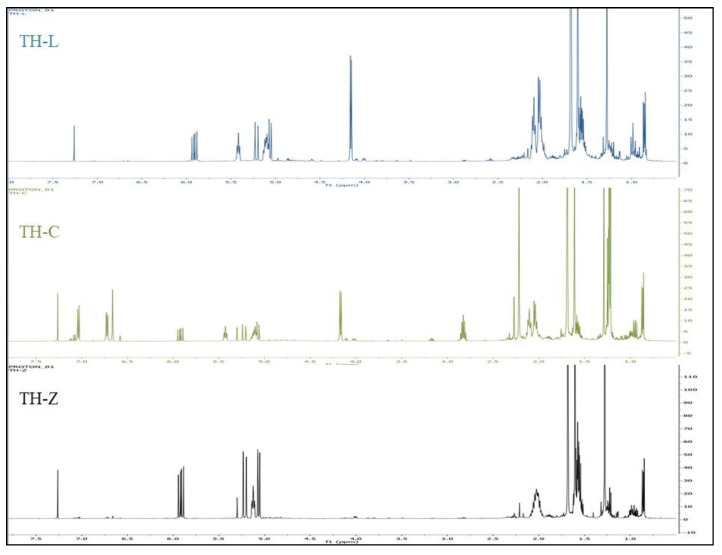
^1^H-NMR from the analysis of the essential oils of *Thymus holosericeus* collected from three Ionian Islands, Greece (TH-Z: Zakynthos; TH-C: Cephalonia, TH-L: Lefkada) during full-flowering (July 2018).

**Figure 3:**
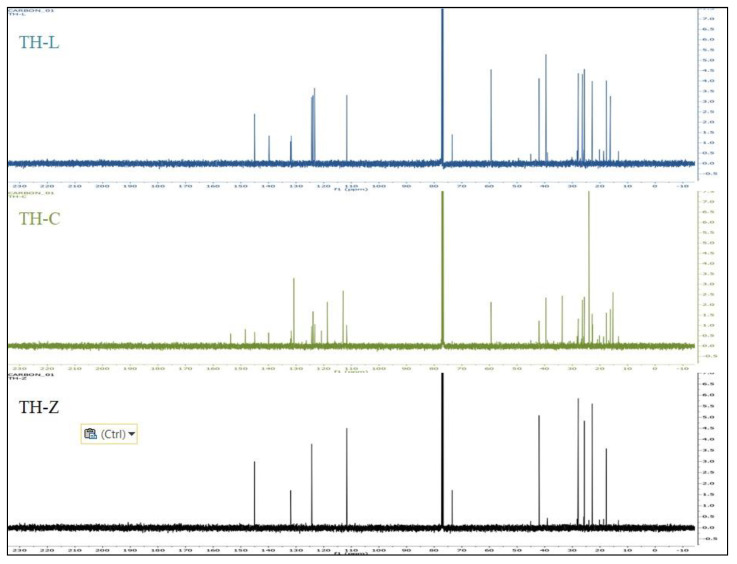
^13^C-NMR from the analysis of the essential oils of *Thymus holosericeus* collected from three Ionian Islands, Greece (TH-Z: Zakynthos; TH-C: Cephalonia, TH-L: Lefkada) during full-flowering (July 2018).

Identification of the main compounds was first performed by the analysis of the ^1^H and ^13^C-NMR spectrum of the total EO of *T. holosericeus* by comparing the signals obtained with those of pure main compounds, and in comparison with the signals in ^1^H and ^13^C-NMR spectra of the mixtures with those of reference spectra (Figure 1, Figure 2 and Figure 3; Supplementary Material Table S1, Supplementary Material Figures S1 and S2). Each compound was unambiguously identified by taking into account the number of identified carbons, the number of overlapped signals and the difference of chemical shifts of each resonance in the mixture spectrum and in the reference. The ^1^H-NMR and ^13^C-NMR analysis of the isolated EOs (Figure 2 and Figure 3) confirm the data obtained from the GC-MS analysis (Table 1). The characteristic signals of the main compounds’ protons appear in the spectra. The comparisons between the recorded spectra are evidence of the different complexes of each EO.

### 2.3. Thin-Layer Chromatography (TLC) Analysis of the Studied Essential Oils

**Figure 4:**
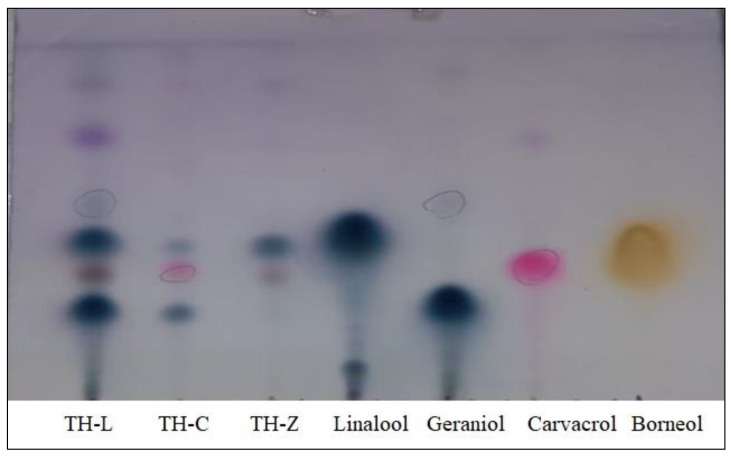
TLC plate analysis of the essential oils of *Thymus holosericeus* from three Ionian Islands, Greece (TH-Z: Zakynthos; TH-C: Cephalonia, TH-L: Lefkada) compared to the standard of the main compounds.

The TLC-fingerprint analysis first performed herein for *T. holosericeus* EOs (Figure 4) confirmed the data obtained from the GC-MS (Table 1). TLC revealed that linalool was one of the few common compounds detected in every EO hydrodistilled from the aerial parts of *T. holosericeus*. Although the sample ordinating from Cephalonia Island was the most abundant in chemical constituents according to the GC-MS analysis, the TLC plate showed that the sample from Lefkada was the most complex one. The main compounds detected in each EO were also visible in the TLC plate. It was obvious that the complexity of the EOs differed greatly.

### 2.4. Ecological Profiling of Natural Preferences

**Figure 5:**
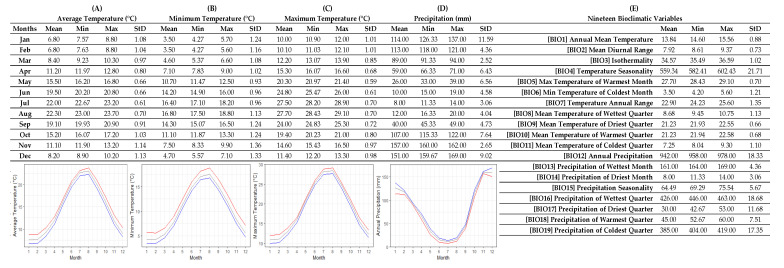
R-derived ecological profile of the wild-growing populations of *Thymus holosericeus* in Cephalonia, Zakynthos and Lefkada islands, Ionian Sea, Greece. In each case (**A**–**E**), the minimum, maximum, average, and standard deviation is shown. Line graphs (**A**–**C**) illustrate the minimum (blue lines), maximum (red lines), and mean (grey lines) monthly values for temperature (°C) and the line graph (**D**) illustrates the minimum (red lines), maximum (blue lines), and mean (grey lines) monthly values for precipitation (mm). (**A**) Minimum temperatures per month (°C), (**B**) maximum temperatures per month (°C), (**C**) average temperatures per month (°C), (**D**) precipitation per month (mm), (**E**) values for 19 bioclimatic variables (Supplementary Material Table S1). All data were extracted from WorldClim version 2.1.

Figure 5 presents the ecological profile of *T. holosericeus* as generated in R. In Figure 5, the highest and lowest values for the monthly temperature and precipitation are marked in red (highest) and blue (lowest), respectively. In the temperature graphs and the precipitation graphs, the highest mean values are marked in red, the average means in grey, and the lowest means in blue, respectively. Similarly, the mean, lowest and highest values for all the 19 bioclimatic variables are also included in the factsheet.

Based on the three available localities sampled in the wild, *T. holosericeus* seems to prefer to thrive in areas with a mean annual temperature of 13.84 ± 0.88 °C and a mean annual precipitation of 942 ± 18.33 mm. The highest temperatures were observed in August (27.70 ± 0.70 °C) and July (27.50 ± 0.70 °C), with the highest value of 29.10 °C. The lowest temperatures were observed in January (6.80 ± 1.08 °C) and February (6.80 ± 1.04 °C), with the lowest value of 7.57 °C in January. These values represent the natural temperature limits occurring in the wild habitats of *T. holosericeus*. The mean precipitation of the wettest quarter was 426 ± 18.68 mm and the mean precipitation of the driest quarter was 30 ± 11.68 mm. The highest mean precipitation was observed in November (157 ± 2.65 mm) but the highest value was observed in December (169 mm), while the lowest mean precipitation and lowest value observed in July (8 ± 3.06 mm and 11 mm, respectively). These values represent the natural precipitation regime prevailing in the wild habitats of *T. holosericeus* (Figure 5).

Apart from the ecological profile of *T. holosericeus*, we compiled the individual climate data for the three sampled areas (TH-C, TH-Z and TH-L) that have been derived from WorldClim version 2.1 to identify differences between them. We further organized the values representing the 19 bioclimatic variables in Table 2, while in Supplementary Material Table S2 we have included detailed values for monthly precipitation and temperature data. Out of the three studied samples, TH-C appears to be characterized by the lowest values for the mean temperature of the driest and warmest quarter, the lowest precipitation in the warmest quarter as well as the lowest average and highest temperatures for the longest part of the year. Therefore, TH-C compared to the other two samples differed from TH-Z and TH-L as the plant material was collected from an area with the driest summer and coldest winter during the year (Table 2).

Similarly, the collected material of TH-Z was characterized by the lowest values for the mean diurnal range, annual precipitation, precipitation of the driest month and driest quarter, and the lowest precipitation in all months of the year, except January and December when it had the highest precipitation among all samples. TH-Z was also characterized by the highest average annual temperature, the highest precipitation on the coldest quarter, and the highest values for the lowest monthly temperatures throughout the year. Thus, the TH-Z plant material was comparatively adapted on more complex conditions with low precipitation during the year, high precipitation only in January and December, higher average temperatures throughout the year, and the lowest mean diurnal range (Table 2).

TH-L plant material was characterized by the highest mean diurnal range, the highest temperature seasonality, the highest temperature value among the three samples, as well as the lowest precipitation during the wettest and warmest quarters. TH-L differed from the other two samples because it was adapted on the highest summer temperatures, highest diurnal range, and lowest precipitation during the warmest and the wettest quarters, but most of the TH-L values were very close to other samples (Table 2).

Although the collected samples of *T. holosericeus* were adapted in different areas and climatic conditions, isothermality, the annual temperature range and mean highest temperatures were relatively similar in all three samples, thus probably representing the basic natural ecological preferences of *T. holosericeus* as a species.

### 2.5. Correlations of Climatic Data and Major Compounds of Essential Oil Composition

The results of the Canonical Correlation Analysis that associate the calculated environmental conditions with the major compounds of the EO chemical composition of *T. holosericeus* (Table 1) are shown and commented in detail in the Supplementary Material Table S3. The significant correlations are summarized right after (high correlations had *p* < 0.005 and very high correlations had *p* < 0.001). Regarding the dominant major compounds characterizing the essential oil of *T. holosericeus* (Table 1 and Supplementary Material Table S3), CCA showed that linalool showed a high positive correlation with the mean annual temperature, mean average temperature in March, April, and September, as well as the highest temperature in January and February. This dominant compound revealed great differences in the concentrations between the samples and CCA showed that its presence is mostly linked to other external factors. CCA results outlined that the dominant major compounds thymol and carvacrol appearing in all samples showed no significant correlations with any of the environmental factors, but their concentrations were significantly higher in TH-C, so they were also probably related to Cephalonia’s dry summers and cold winters with low temperatures. Borneol across samples showed a high negative correlation with the mean diurnal range, isothermality, high positive correlation for precipitation of the coldest quarter, precipitation in January and October; and very high positive correlation for the precipitation of the wettest quarter. The dominant major compound geraniol that only appeared in TH-C and TH-L had a very high correlation with all environmental factors except for the lowest temperature which was the same for both samples. The correlations were negative with the annual precipitation, precipitation of the wettest month, precipitation seasonality, precipitation of wettest quarter, precipitation of coldest quarter, and precipitation from October until April and the rest of the correlations were negative.

Some other dominant compounds appearing in two of the samples (Table 1 and Supplementary Material Table S3) were *p*-cymene, which, similarly to *α*-terpinene, only appeared in TH-Z and TH-C samples (considerably higher concentration in TH-C) and showed very high correlation with all environmental variables. The correlation was positive for the mean diurnal range, isothermality, temperature seasonality, temperature annual range, annual precipitation, precipitation of the driest month and driest quarter, precipitation in September and from February to August, while the rest of the environmental factors had a negative correlation on *p*-cymene. Geranial that only appeared in TH-C and TH-L had a very high correlation with all environmental factors, except for the lowest temperature which was the same for both samples. The correlations were negative with the annual precipitation, precipitation of the wettest month, precipitation seasonality, precipitation of the wettest quarter, precipitation of the coldest quarter, precipitation from October until April and the lowest temperature in November and December. The rest of the correlations were negative. Geranial showed the same significant correlations with neral except for the lowest temperatures for November and December that were also negative.

Some of the compounds appeared in all samples (Table 1 and Supplementary Materials Table S3), such as camphor, which was detected in Zakynthos and Lefkada, but traces were also identified in the sample from Cephalonia. Camphor showed a very high correlation with all environmental factors. The correlation was positive with mean diurnal range, isothermality, temperature seasonality, the highest temperature in the warmest month, lowest temperature in the coldest month, annual temperature range, annual precipitation, precipitation in the driest month and quarter, precipitation from January until September and in November, and highest temperature from May until August. It is suggested that this compound is likely related to the prevailing precipitation conditions in Lefkada since TH-L’s concentration was significantly higher than in TH-Z. Terpinen-4-ol showed a high negative correlation for the highest temperature in July, and it is also a compound with the highest concentration in TH-C which is likely related to Cephalonia’s special environment. Just like *α*-thujene, *β*-pinene, limonene and *γ*-terpinene. *β*-Caryophyllene showed a high positive correlation with the precipitation of the warmest quarter, August’s average temperature, as well as March and October’s highest temperatures. This compound had high concentrations of TH-Z and TH-L but a considerably lower concentration of TH-C. Caryophyllene oxide did not show any significant correlation with any of the environmental factors. This compound had high concentrations of TH-Z and TH-L but a lower concentration of TH-C. It is assumed that this compound is affected by external factors.

Some of the main compounds appeared in only one of the samples (Table 1 and Supplementary Materials Table S3) and CCA showed that *γ*-terpinene and geranyl acetate found in TH-C and TH-L, respectively, had no significant correlation with any of the environmental factors studied. These are probably related with the special combination of climatic conditions prevailing in each of the Ionian islands.

Although the minor compounds detected in the samples may not directly affect the samples’ chemotypes, the CCA of many minor compounds showed a close association with the local environment of each sample (detailed results in the Supplementary Materials Table S3).

Essential oils are not stable in terms of quality and quantity, and they adapt accordingly to biological interactions, environmental challenges and survival needs [11,31,32]. Plants with complex essential oil profiling, such as those sampled in Zakynthos (TH-Z) can probably adapt more easily to a local environment with harsher climate conditions, like Zakynthos, which showed the highest temperature value paired to the least precipitation for the longest time during the year. This regime was directly related to significantly higher concentrations of a higher number of compounds in TH-Z EO compared to the other two samples. Additionally, TH-L from Lefkada have showcased less complex environmental conditions since many chemical compounds that were found in TH-Z and TH-C were not detected in TH-L. It has to be highlighted that some chemical compounds that were identified in TH-L were absent from TH-C, which also denotes that some chemical compounds are probably induced under less complex and/or different environmental conditions.

Although chemotypes and related environmental factors have been studied before in other *Thymus* spp. such as *T. migricus* Klokov & Des.-Shost. [33] showcasing the impact of climatic variability on the qualitative and quantitative properties of wild-growing populations, it is the first time that *T. holosericeus* has been approached from such a viewpoint. Generally, it is known that some compounds seem to be induced from natural external factors and others comprise inherited genetic factors [34,35]. In this study, we only analyzed data from WorldClim.org and not samples of natural soils or other external factors, since the soil conditions were similar to all three sampled sites of the Ionian Islands. Undoubtedly, a different research approach would be necessary to identify other external factors that may influence extant *T. holosericeus* chemotypes detected in the wild. We herein suggest a larger scale of targeted studies to accurately analyze *T. holosericeus* chemotypes across its range in the Ionian archipelago in an attempt to provide more detailed insight regarding the environmental or external factors that affect its natural essential oil composition. When performed, the management of its natural production potential can be further assessed in man-made settings. Additionally, this research line combined with extant knowledge on species-specific propagation [10,13] and the ecological profiling outlined herein can also facilitate the sustainable exploitation of the endangered *T. holosericeus* as a new thyme crop [14,15,16], and may help to mitigate its possible overharvesting from the wild [33]. Additionally, the ecological profile of *T. holosericeus* as reported herein can be further used to inform and guide species-specific in situ and ex situ conservation efforts and sustainable exploitation strategies [36,37].

## 3. Materials and Methods

### 3.1. Collections of Plant Material

**Figure 6:**
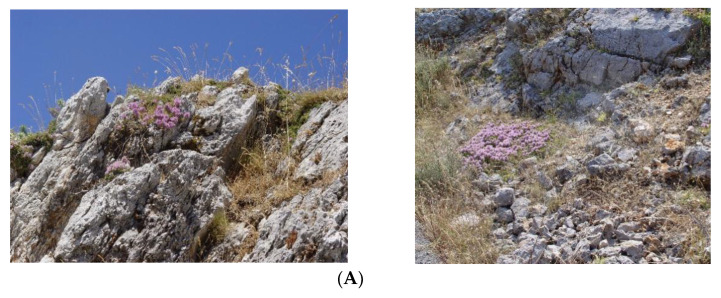

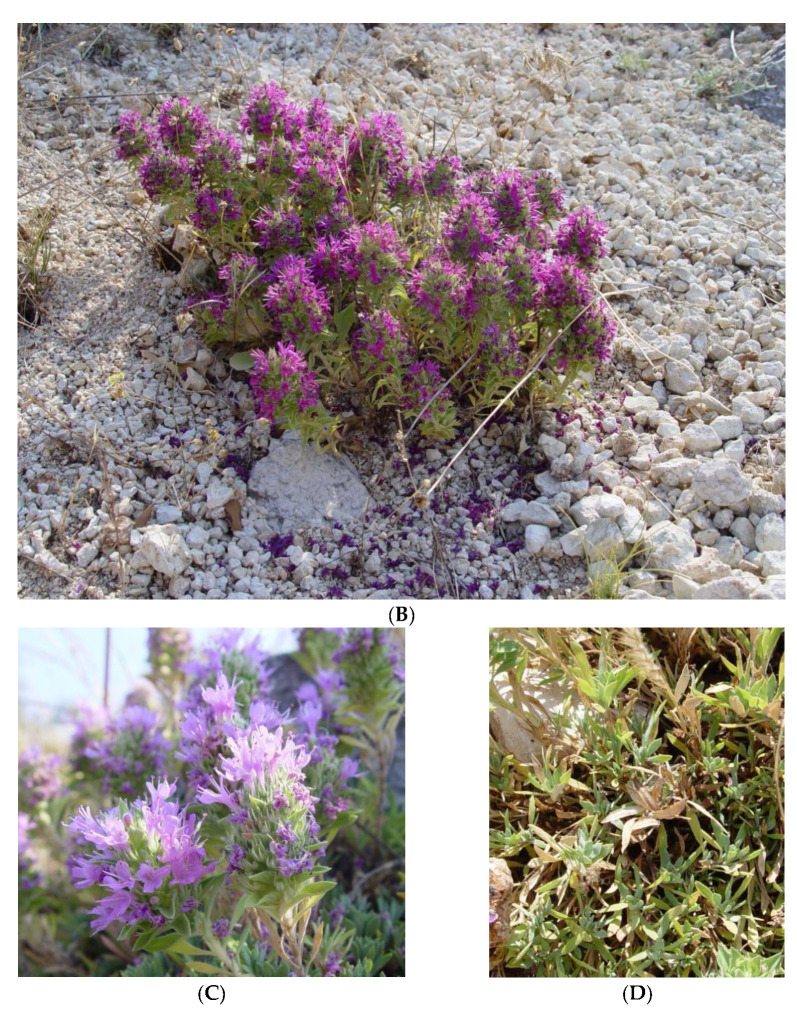
Representative illustration of *Thymus holosericeus* sampled from wild habitats in Cephalonia (**A**) and Lefkada (**B**) Islands (Greece) with variations in flower color and inflorescences (**B**,**C**) and typical leaves (**B**,**D**).

Aerial parts of *T. holosericeus* (Figure 6) were collected randomly by hand from 10 wild-growing plant individuals thriving in each of the three different Ionian Islands (Table 3) during their full-flowering period (July 2018), that is, ten individuals from Orthonies-Volimes in Zakynthos Island (TH-Z), another ten from Mt Roudi, Cephalonia Island (TH-C), and another ten from Mt Elati in Lefkada Island (TH-L). The authorized collections of plant material were performed using the official permit of the Institute of Plant Breeding and Phytogenetic Resources, Hellenic Agricultural Organization Demeter, which is issued yearly by the Greek Ministry of Environment and Energy (e.g., Permit 82336/879 of 18 May 2019 and 26895/1527 of 21 April 2021). The harvested plants were taxonomically identified by Dr. Nikos Krigas and reference materials are maintained under ex situ conservation with the following International Plant Exchange Network (IPEN) accession numbers: GR-1-BBGK-08,4583 for TH-L, GR-1-BBGK-0805,2875 and GR-1-BBGK-08,4957 for TH-C, and GR-1-BBGK-09,5403 for TH-Z. The respective voucher specimens with the same IPEN accession numbers have been deposited at the Herbarium of the Balkan Botanic Garden of Kroussia (acronym BBGK according to Index Herbariorum) with duplicates kept at the School of Pharmacy, Aristotle University of Thessaloniki (Greece).

### 3.2. Sample Preparation

The collected aerial parts of *T. holosericeus* were air-dried at room temperature (~25 °C) for 10 days in a shaded and well-ventilated place, then were ground to fine powder and stored in a shaded place. Several hydrodistilations (each sample was extracted three times) were prepared from the initial material (TH-Z: 41.5 g, TH-C: 48.2 g, TH-L: 41.6 g) using a Clevenger apparatus according to standard procedures [38]. The volatiles were trapped in 5 mL GC-grade n-pentane according to a standard procedure (European Pharmacopoeia, 2005), dried over anhydrous sodium sulfate, and kept in closed, air-tight Pyrex containers at −4 °C. The essential oil (EO) yield was expressed in mL 100^−1^ g d.w.

### 3.3. Gas Chromatography-Mass Spectrometry Analysis

Essential oil (EO) analyses were performed using previously described methods [39]. These analyses were performed on a Shimadzu GC-2010-GCMS-QP2010 system operating at 70 eV equipped with a split/splitless injector (230 °C) and a fused silica HP-5 MS capillary column (30 m × 0.25 mm i.d., film thickness 0.25 μm). The temperature program was set from 50 °C to 290 °C, at a rate of 4 °C/min. Helium was used as a carrier gas at a flow rate of 1.0 mL/min. The injection volume of each sample was 1.0 μL. Retention indices for all compounds were determined according to the Ref. [40], using n-alkanes as standards. The identification of the components was based on a comparison of their mass spectra with those of NIST21 and NIST107 [41], and by comparison of their retention indices with literature data [42]. The essential oils obtained were subjected to co-chromatography with authentic compounds (Fluka, Sigma-Aldrich, Darmstadt, Germany).

### 3.4. Nuclear Magnetic Resonance Analysis

^1^H-NMR and 1^3^C-NMR spectra were obtained on AGILENT DD2 500 [500 MHz (^1^H) 125.5 MHz (^13^C)]. Chemical shifts are reported in δ (ppm) values, relative to TMS (7.26 ppm for ^1^H-NMR and 77.00 ppm for ^13^C-NMR for CDCl_3_). In total, 50 μL of each essential oil sample and high-purity standard monoterpenes (linalool, borneol, geraniol and carvacrol) were diluted with deuterated chloroform.

### 3.5. Thin-Layer Chromatography (TLC) Analysis

Chromatographic separation was carried out on silica gel (Kieselgel F254, Merck, Art. 5554) chromatographic plates of 10 cm × 10 cm with 0.1 mm thickness. The EOs as well as the standard main compounds were dissolved in a mixture of dichloromethane: hexane (90:10) and were spotted on the chromatographic plates, then developed using a mixture of n-hexane and acetone (90:10, *v*/*v*). Detection on TLC plates: UV light (absorbance: 254 and 366 nm), vanillin-H_2_SO_4_ spray reagent on silica gel.

### 3.6. Ecological Profiling of T. holosericeus

The ecological profiling was performed with R (R Core Team, 2013), following a previously published methodology [43,44] for ecological profiling with Geographic Information Systems (GIS). In short, we collected climate data for 19 bioclimatic variables as well as monthly data for temperature and precipitation [43,44] regarding the three sampling locations of *T. holosericeus* (TH-C, TH-Z, TH-L) from the online database of WorldClim [45]. In this study, we utilized WorldClim version 2.1 which includes mean values of climate data from 1970 to 2000 and are widely used [45]. We used R’s raster package [44] to associate the three locations with a stack of the WorldClim’s 19 bioclimatic information (Supplementary Material 2) and extract arithmetic values to present and organize these data on a single factsheet (e.g., [43,44]). The final factsheet (Figure 5) summarizes and graphically visualizes the calculated mean values and graphs for the monthly Minimum Temperature (Tmin), mean values and graphs for the monthly Maximum Temperature (Tmax), mean values and graphs for the monthly Annual Precipitation (Pannual) and the mean values of the 19 bioclimatic variables associated with the collection locations of *T. holosericeus* [43,44], thus providing an insight into the basic ecological preferences of the studied species.

Additionally, the climate information that was extracted separately [45] for each one of the three collection sites have been compiled in Table 2 to compare and identify the differences in the climate conditions between the three wild locations (TH-C, TH-Z and TH-L).

### 3.7. Canonical Correlation Analysis (CCA)

Furthermore, the data of the generated ecological profile and the data of the examined essential oils were statistically analyzed with Pearson’s Canonical Correlation Analysis (CCA) to identify possible correlations between the prevailing environmental conditions (including monthly temperature and precipitation data and the 19 bioclimatic variables) and the essential oil composition of the studied samples (including 40 phytochemical compounds) by determining canonical variates [46,47]. The results of the CCA have been compiled in the Supplementary Materials Table S3 for all the compounds found in the essential oil composition of the three samples of *T. holosericeus*.

## 4. Conclusions

The results reported herein may serve the complex chemotaxonomy of the taxa in genus *Thymus* and the investigation of chemotypes of *T. holosericeus* in refined geographical scales (different islands in an archipelago) providing evidence regarding the correlations of essential oil compounds and climatic attributes. This study revealed the complexity and the natural variation of the essential oils of a single range-restricted and taxonomically isolated local endemic thyme species (*Thymus holesericeus*) based on plant material collected during the same period from three nearby islands which belong to the same geographical zone (archipelago of Ionian Islands) and identified patterns of variation related with climatic attributes. The results of the studied essential oils were documented by nuclear magnetic resonance analysis and TLC analysis and offer support to the existence of different chemotypes in *T. holosericeus*. Although a pure linalool chemotype was naturally found, we also documented mixed-type chemotypes (carvacrol/geraniol or linalool/geraniol chemotypes). Only few compounds were detected in considerable quantity in every sample, and among them were linalool (14.37–82.77%), *β*-Caryophyllene (1.83–2.87%) and Borneol (5.20–5.95%), while oxygenated monoterpenes were the major constituents in all samples. Although the quantity of samples that have been analyzed may seem insufficient, we must highlight that there are only 12 known locations in which *T. holoserieceus* has been recorded to date, and therefore, our study has focused on 25% of the globally available species population, including the major ones. The ecological and essential oil profiling of *T. holosericeus* as reported herein may facilitate its sustainable exploitation as a new medicinal and aromatic crop, namely, a local Greek endemic thyme species. The qualitative and qualitative characteristics of the studied essential oils of *T. holesericeus* were shown to be closely related to specific environmental factors which resulted in different wild-growing populations having a special profile of essential oils due to their location in which they thrive, and their adaptability to local environmental stress factors. This natural chemical variability of the essential oils of the Ionian thyme is an important asset to be mastered in man-made agricultural settings and it could play a key role in future artificial selection strategies and breeding programmes.

## Figures and Tables

**Table 1:** Composition of the essential oils of *Thymus holosericeus* collected from three Ionian Islands, Greece (TH-Z: Zakynthos; TH-C: Cephalonia, TH-L: Lefkada) during full-flowering (July 2018). The dominant compounds at least in one sample are marked in bold.

				Ionian Islands			
				Zakynthos	Cephalonia	Lefkada	
**Total Yield**				1.93% ± 0.07	2.28% ± 0.12	1.92% ±0.09	
**^a^ Compounds**		**^b^** **RI_exp_**	**RI_lit_**	**TH-Z**	**TH-C**	**TH-L**	**^c^ ID**
1	*α*-Thujene	926	924	nd	0.13 ± 0.01	nd	RI, MS
2	*α*-Pinene	932	932	0.18 ± 0.01	0.22 ± 0.01	0.14 ± 0.01	RI, MS, Co-GC
3	Camphene	946	946	0.39 ± 0.09	0.36 ± 0.11	0.32 ± 0.05	RI, MS
4	*β*-Pinene	974	974	nd	Tr	nd	RI, MS, Co-GC
5	1-Octen-3-ol	978	974	0.34 ± 0.11	0.34 ± 0.23	0.18 ± 0.06	RI, MS
6	Myrcene	991	988	0.10 ± 0.01	0.15 ± 0.01	0.07 ± 0.00	RI, MS, Co-GC
7	*α*-Terpinene	1015	1014	0.15 ± 0.01	0.47 ± 0.01	nd	RI, MS, Co-GC
8	***p*-Cymene**	1024	1020	0.32 ± 0.01	**4.08 ± 0.01**	nd	RI, MS, Co-GC
9	Limonene	1027	1024	nd	0.39 ± 0.08	nd	RI, MS, Co-GC
10	1,8-Cineole	1030	1026	0.21 ± 0.03	nd	0.11 ± 0.01	RI, MS, Co-GC
11	***γ*-Terpinene**	1058	1054	0.32 ± 0.07	**1.05** ± 0.22	0.06 ± 0.02	RI, MS, Co-GC
12	*cis*-Sabinene hydrate	1068	1065	0.25 ± 0.08	nd	nd	RI, MS
13	*cis*-Linalool oxide (furanoid)	1073	1067	0.44 ± 0.10	0.12 ± 0.00	0.49 ± 0.01	RI, MS
14	*trans*-Linalool oxide(furanoid)	1087	1084	0.56 ± 0.0	0.22 ± 0.03	0.50 ± 0.06	RI, MS
15	**Linalool**	1099	1095	**82.77 ± 0.93**	**14.37 ± 0.08**	**40.37± 0.10**	RI, MS, Co-GC
16	Camphor	1144	1141	0.10 ± 0.00	Tr	**2.03 ± 0.01**	RI, MS, Co-GC
17	**Borneol**	1165	1165	**5.95 ± 0.22**	**5.66 ± 0.17**	**5.20 ± 0.09**	RI, MS, Co-GC
18	**Terpinen-4-ol**	1177	1174	**1.61** ± 0.25	**2.43** ± 0.06	0.69 ± 0.08	RI, MS, Co-GC
19	*α*-Terpineol	1190	1186	0.19 ± 0.87	0.21 ± 0.02	0.15 ± 0.07	RI, MS, Co-GC
20	*trans*-Dihydro carvone	1203	1200	nd	0.10 ± 0.01	nd	RI, MS
21	Nerol	1229	1227	nd	0.89 ± 0.04	0.74 ± 1.63	RI, MS, Co-GC
22	Neral	1240	1235	nd	0.22 ± 0.02	0.64 ± 0.08	RI, MS
23	Carvone	1244	1239	nd	0.38 ± 0.02	nd	RI, MS
24	Thymoquinone	1251	1248	nd	0.07 ± 0.00	nd	RI, MS
25	**Geraniol**	1255	1249	nd	**23.98 ± 1.24**	**39.42 ± 0.86**	RI, MS
26	Geranial	1272	1264	nd	0.75 ± 0.02	**1.10 ± 0.21**	RI, MS
27	Bornyl acetate	1286	1287	nd	0.12 ± 0.00	0.0 8± 0.01	RI, MS, Co-GC
28	**Thymol**	1291	1289	0.31 ± 0.02	**4.27** ± 1.07	0.04 ± 0.01	RI, MS, Co-GC
29	**Carvacrol**	1300	1298	**1.07** ± 0.03	**35.34** ± 0.87	0.28 ± 0.00	RI, MS
30	Geranyl acetate	1383	1379	nd	nd	**1.00** ± 0.30	RI, MS
31	*β*-Caryophyllene	1420	1408	**2.87** ± 0.19	1.83 ± 1.56	**2.36** ± 0.44	RI, MS, Co-GC
32	*β*-Copaene	1431	1430	nd	nd	0.04 ± 0.02	RI, MS
33	*α*-Humulene	1455	1452	nd	nd	0.08 ± 0.01	RI, MS, Co-GC
34	Germacrene D	1483	1484	0.18 ± 0.00	0.05 ± 0.01	0.32 ± 0.03	RI, MS, Co-GC
35	Bicyclogermacrene	1498	1500	nd	nd	0.10 ± 0.00	RI, MS
36	Geranyl isobutanoate	1515	1514	nd	nd	0.12 ± 0.00	RI, MS
37	Geranyl butanoate	1562	1562	nd	nd	0.26 ± 0.01	RI, MS
38	Spathulenol	1580	1577	nd	0.25 ± 0.02	0.11 ± 0.02	RI, MS
39	**Caryophyllene oxide**	1585	1582	0.78 ± 0.02	0.93 ± 0.11	**2.16** ± 0.01	RI, MS, Co-GC
40	Caryophylla-4(12),8(13)-dien-5-ol	1640	1639	nd	nd	0.14 ± 0.00	RI, MS
Total %				99.09	99.38	99.30	
Monoterpene Hydrocarbons				1.46	6.85	0.84	
Oxygenated Monoterpenes				92.08	40.40	88.40	
Aromatic compounds				1.38	39.61	0.32	
Sesquiterpene Hydrocarbons				3.05	1.88	2.90	
Oxygenated Sesquiterpenes				0.78	1.18	2.41	

^a^ The compounds detected are listed in order of elution from an HP-5 MS capillary column. ^b^ RI: Retention indices as determined on a HP-5 MS capillary column using a homologous series of n-alkanes (C9-C25), RIexp = RI experimental, RIlit = RI literature; ^c^ ID (Identification method): RI = Retention Index, MS = Mass Spectrum, Co-GC: co-injection with authentic compound; nd = not detected. Concentrations below 0.05% are marked as tr (traces).

**Table 2:** Climate data (19 bioclimatic variables and units) for the three sampled areas of *Thymus holocericeus* in Cephalonia (TH-C), Zakynthos (TH-Z), and Lefkada (TH-L) islands, Ionian Sea, Greece derived from WorldClim version 2.1.

Data for 19 Bioclimatic Variables per Sampled Population of *Thymus holosericeus*			
	TH-C	TH-Z	TH-L
Annual Mean Temperature (°C)	13.84	15.56	14.40
Mean Diurnal Range (°C)	8.54	7.92	9.37
Isothermality (%)	35.30	34.57	36.59
Temperature Seasonality (%)	585.46	559.34	602.43
Max Temperature of Warmest Month (°C)	27.70	28.50	29.10
Min Temperature of Coldest Month (°C)	3.50	5.60	3.50
Temperature Annual Range (°C)	24.20	22.90	25.60
Mean Temperature of Wettest Quarter (°C)	8.68	10.75	8.92
Mean Temperature of Driest Quarter (°C)	21.23	22.55	22.02
Mean Temperature of Warmest Quarter (°C)	21.23	22.58	22.02
Mean Temperature of Coldest Quarter (°C)	7.25	9.30	7.57
Annual Precipitation (mm)	978.00	942.00	954.00
Precipitation of Wettest Month (mm)	162.00	169.00	161.00
Precipitation of Driest Month (mm)	12.00	8.00	14.00
Precipitation Seasonality (%)	67.85	75.54	64.49
Precipitation of Wettest Quarter (mm)	449.00	463.00	426.00
Precipitation of Driest Quarter (mm)	45.00	30.00	53.00
Precipitation of Warmest Quarter (mm)	45.00	60.00	53.00
Precipitation of Coldest Quarter (mm)	408.00	419.00	385.00

**Table 3:** Localities collection of *Thymus holosericeus* from three Ionian Islands (Greece) recorded in the WGS84 coordinate system.

Ionian Island	Area	Altitude (m)	Latitude (North)	Longitude (East)
Cephalonia	Mt Roudi	565	38°11′915″	20°35′86″
Lefkada	Mt Elati, Hortata,1.7 km before Asprogerakata	786	38°46′55″	20°29′10″
Zakynthos	2.4 km NW of Orthonies to Volimes	350	37°45′00″	20°55′00″

## Data Availability

All data referred to or generated in this study are included in the Tables, Figures and Supplementary Materials and are available upon request.

## Supplementary Materials

The following supporting information can be downloaded at: https://www.mdpi.com/article/10.3390/plants12020348/s1, Table S1: Literature values of the main compounds and observed values of ^1^H-NMR in the essential oils of *T. holosericeus* from three Ionian Islands, Greece (TH-Z: Zakynthos; TH-C: Cephalonia, TH-L: Lefkada); Figure S1: ^1^H-NMR from the analysis of the essential oils of *T. holosericeus* from three Ionian Islands, Greece (TH-Z: Zakynthos; TH-L: Lefkada; TH-C: Cephalonia) compared to the ^1^H-NMR of the standard of their main compounds; Figure S2: ^13^C-NMR from the analysis of the essential oils of *T. holosericeus* from three Ionian Islands, Greece (TH-Z: Zakynthos; TH-L: Lefkada; TH-C: Cephalonia) compared to the ^13^C-NMR of the standard of their main compounds; Table S2: Detailed monthly climate data for the three sampled areas of *Thymus holocericeus* in Cephalonia (TH-C), Zakynthos (TH-Z), and Lefkada (TH-L) islands, Ionian Sea, Greece derived in R from WorldClim version 2.1; Table S3: Pearson’s Canonical Correlation Analysis calculated in SPSS for all compounds and the 19 bioclimatic variables, the monthly precipitation, the monthly average temperature, the monthly highest temperature and the monthly lowest temperature. References [48,49,50] are cited in the Supplementary Materials.
