# Supplementary material for: Exploring the Ecological Preferences and Essential Oil Variability in Wild-Growing Populations of the Endangered Local Greek Endemic Thymus holosericeus (Lamiaceae)
Source: Plants (Basel). 2023 Jan 11;12(2):348. doi: 10.3390/plants12020348 (PMC9864777; doi:10.3390/plants12020348)
Supplement: Supplementary file 1 [file plants-12-00348-s001.zip › _Supplementary Material S1.pdf]

Supplementary Material

Table S1: Literature values of the main compounds and observed values of ^{1}H-NMR in the essential oils of *Thymus holosericeus* from three Ionian Islands, Greece (TH-Z: Zakynthos; TH-C: Cephalonia, TH-L: Lefkada)

	**Literature values (δ)**					
	Linalool [48]		Carvacrol [49]		Geraniol [50]	
	H	C	H	C	H	C
1	5.33 d (2H)	120.17	-	153.6	4.154 d (J=6.9Hz) 1H	59.5
2	5.91 t 1H	145.25	-	126.9	5.41 m 1H	123.4
3	-	75.68	7.08 d (J=5Hz)	130.9	-	139.9
4	1.37 2H	43.87	6.78 d (J=5Hz)	118.8	o.s. 1.98-2.16	26.5
5	2.01q 2H	22.52	-	148.5	o.s. 1.98-2.16	39.6
6	5.19 t 1H	108.65	6.70 s 1H	113.0	5.09 m 1H	131.9
7	-	131.96	2.26 s 3H	15.4	-	124.0
8	1.63 3H	17.53	2.86 m 3H	33.7	1.68 s 3H	25.8
9	1.76 3H	24.95	1.26 d (J=5Hz)	24.1	1.60 s 3H	16.4
10	0.90 3H	29.8	1.26 d (J=5Hz)	24.1	1.68 s 3H	17.8

**Observed values (δ)**					
TH-Z		TH-C		TH-L	
		7.08 d (J=5Hz)	130.9		
		6.78 d (J=5Hz)	118.8		
		6.70 s 1H	113.0		
5.91 t 1H	145.3	5.91 t 1H	145.3	5.91 t 1H	145.2
		5.41 m 1H	123.4	5.41 m 1H	123.4
5.33 d 2H	120.2	5.33 d (2H)	120.1	5.33 d 2H	120.2
5.19 t 1H	108.8	5.19 t 1H	108.6	5.19 t 1H	108.7
		5.10 m 1H	131.9	5.10 m 1H	131.9
		4.14 d (J=6.9Hz) 1H	59.3	4.14 d (J=6.9Hz) 1H	59.3
		2.86 m 3H	33.7		
		2.26 s 3H	15.4		
		o.s. 1.98-2.16 4H	39.8	o.s. 1.98-2.16 4H	39.7
			25.4		25.3
2.01q 2H	22.4	2.01q 2H	22.6	2.01q 2H	22.5
1.76 3H	24.8	1.76 3H	24.9	1.76 3H	25.0
		1.68 s 6H	25.7	1.68 s 6H	25.9
			17.9		18.0
1.63 s 3H	17.4	1.63 3H	17.5	1.63 3H	17.5
		1.60 s 3H	16.4	1.60 s 3H	16.3
1.37 2H	44.0	1.37 2H	43.9	1.37 2H	43.8
		1.26 d (J=5Hz) 6H	24.1		
0.91 s 3H	29.7	0.90 3H	30.0	0.90 3H	29.8

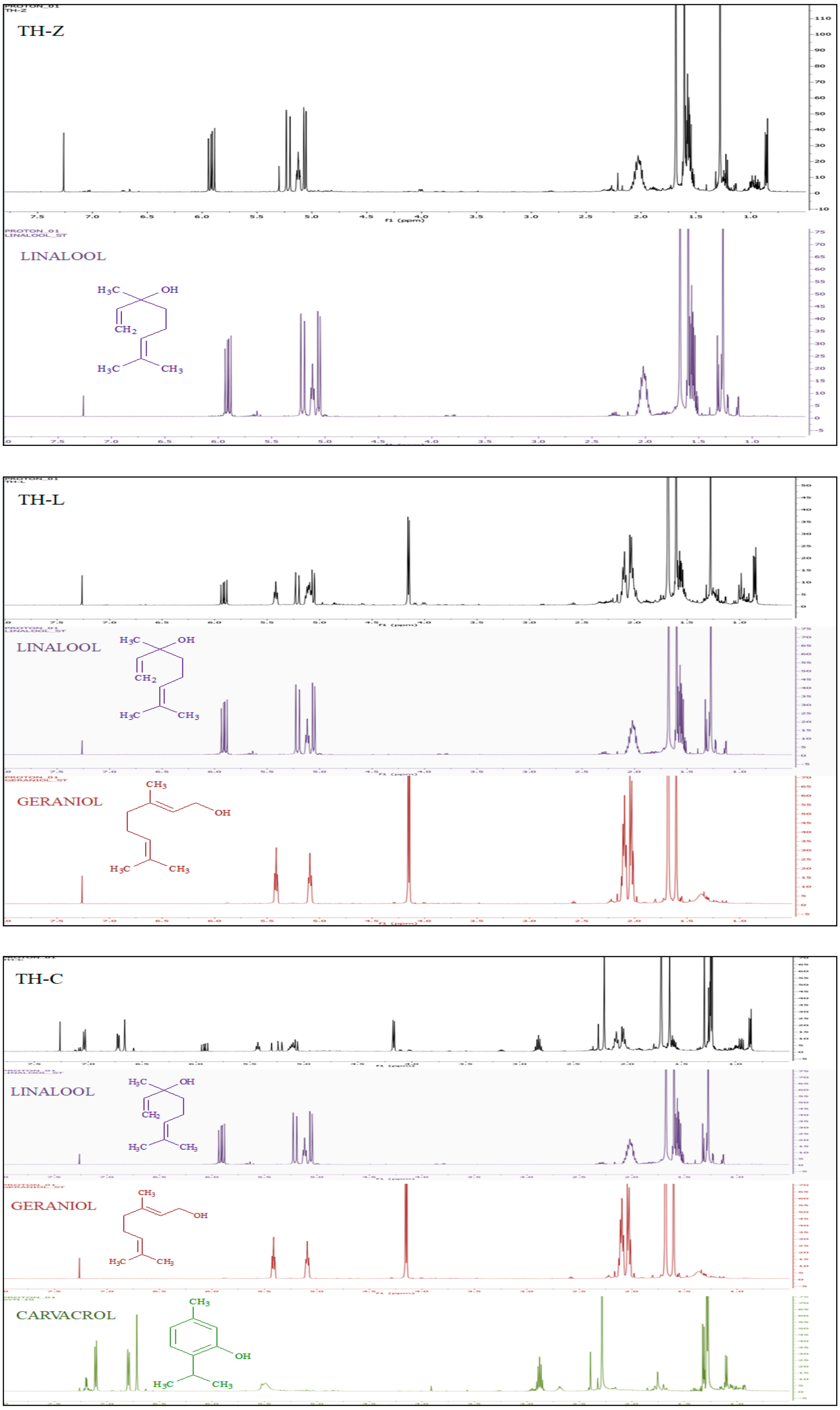

Figure S1: ^{1}H-NMR from the analysis of the essential oils of *T. holosericeus* from three Ionian Islands, Greece (TH-Z: Zakynthos; TH-L: Lefkada; TH-C: Cephalonia) compared to the ^{1}H-NMR of the standard of their main compounds.

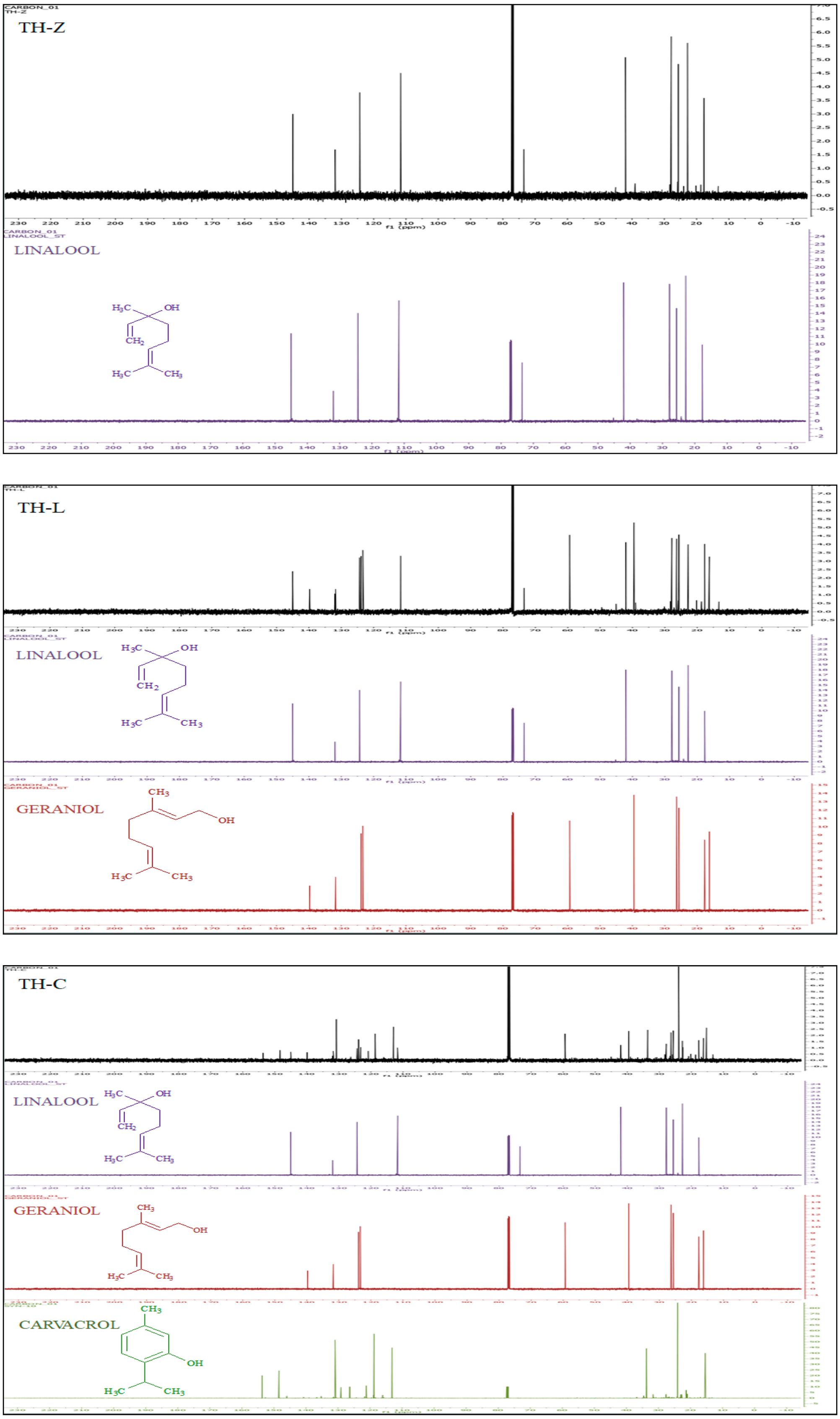

Figure S2: ^{13}C-NMR from the analysis of the essential oils of *T. holosericeus* from three Ionian Islands, Greece (TH-Z: Zakynthos; TH-L: Lefkada; TH-C: Cephalonia) compared to the ^{13}C-NMR of the standard of their main compounds.

References

48. Uttu, A.J.; Sallau, M. S.; Iyun, O. R. A.; Ibrahim, H. Isolation, characterization and *in silico* molecular docking studies of two terpenoids from *Strychnos innocua* Delile root bark for antibacterial properties. *Adv. J. Chem. -Sect. A* **2022**, *5*, 241-252. https://doi.org/10.22034/AJCA.2022.344451.1315

49. Masila, V.M.; Ndakala, A.J.; Midiwo, J.O.; Byamukama, R.; Kamau, R.W.; Kumarihamy, M.; Muhammad, I. Synthesis of a pyrrolidine derivative of a carvotacetone and monoterpenes for anti-methicillin-resistant *Staphylococcus aureus* and anti-cryptococcal properties. *Nat. Prod. Res.* **2022**, *36*, 2321 – 2328. https://doi.org/10.1080/14786419.2020.1833201

50. Zimmermann, B.M.; Ngoc, T.T.; Tzaras, D.I.; Trinadh, K.; Teichert, J.F. Bifunctional copper catalyst enables ester reduction with H_2: Expanding the reactivity space of nucleophilic copper hydrides. *J. Am. Chem. Soc.* **2021**, *143*, 40, 16865–16873. https://doi.org/10.1021/jacs.1c09626
