# Supplementary material for: Exploring the Ecological Preferences and Essential Oil Variability in Wild-Growing Populations of the Endangered Local Greek Endemic Thymus holosericeus (Lamiaceae)
Source: Plants (Basel). 2023 Jan 11;12(2):348. doi: 10.3390/plants12020348 (PMC9864777; doi:10.3390/plants12020348)
Supplement: Supplementary file 1 [file plants-12-00348-s001.zip › _Supplementary Material S2.pdf]

Supplementary Material Table S2. Detailed monthly climate data for the three sampled areas of *Thymus holocericeus* in Cephalonia (TH-C), Zakynthos (TH-Z), and Lefkada (TH-L) islands, Ionian Sea, Greece derived in R from WorldClim version 2.1

**Monthly Climate data per sampled population of *Thymus holosericeus***			
	**TH-C**	**TH-Z**	**TH-L**
Precipitation in January (mm)	128.00	137.00	114.00
Precipitation in February (mm)	121.00	113.00	120.00
Precipitation in March (mm)	94.00	89.00	91.00
Precipitation in April (mm)	71.00	59.00	69.00
Precipitation in May (mm)	34.00	26.00	39.00
Precipitation in June (mm)	16.00	10.00	19.00
Precipitation in July (mm)	12.00	8.00	14.00
Precipitation in August (mm)	17.00	12.00	20.00
Precipitation in September (mm)	47.00	40.00	49.00
Precipitation in October (mm)	117.00	122.00	107.00
Precipitation in November (mm)	162.00	157.00	161.00
Precipitation in December (mm)	159.00	169.00	151.00
Average Temperature in January (^{o}C)	6.80	8.80	7.10
Average Temperature in February (^{o}C)	6.80	8.80	7.30
Average Temperature in March (^{o}C)	8.40	10.30	9.00
Average Temperature in April (^{o}C)	11.20	12.80	11.90
Average Temperature in May (^{o}C)	15.50	16.80	16.30
Average Temperature in June (^{o}C)	19.50	20.80	20.30
Average Temperature in July (^{o}C)	22.00	23.20	22.80
Average Temperature in August (^{o}C)	22.30	23.70	23.00
Average Temperature in September (^{o}C)	19.10	20.90	19.80
Average Temperature in October (^{o}C)	15.20	17.20	15.80
Average Temperature in November (^{o}C)	11.10	13.20	11.40
Average Temperature in December (^{o}C)	8.20	10.20	8.30
Highest Temperature in January (^{o}C)	10.00	12.00	10.70
Highest Temperature in February (^{o}C)	10.10	12.10	10.90
Highest Temperature in March (^{o}C)	12.20	13.90	13.10
Highest Temperature in April (^{o}C)	15.30	16.60	16.30
Highest Temperature in May (^{o}C)	20.30	21.20	21.40
Highest Temperature in June (^{o}C)	24.80	25.60	26.00
Highest Temperature in July (^{o}C)	27.50	28.20	28.90
Highest Temperature in August (^{o}C)	27.70	28.50	29.10
Highest Temperature in September (^{o}C)	24.00	25.30	25.20
Highest Temperature in October (^{o}C)	19.40	21.00	20.30
Highest Temperature in November (^{o}C)	14.60	16.50	15.20
Highest Temperature in December (^{o}C)	11.40	13.30	11.90
Lowest Temperature in January (^{o}C)	3.60	5.70	3.50
Lowest Temperature in February (^{o}C)	3.50	5.60	3.70
Lowest Temperature in March (^{o}C)	4.60	6.60	4.90
Lowest Temperature in April (^{o}C)	7.10	9.00	7.40

Lowest Temperature in May (°C)	10.70	12.50	11.20
Lowest Temperature in June (°C)	14.20	16.00	14.50
Lowest Temperature in July (°C)	16.40	18.20	16.70
Lowest Temperature in August (°C)	16.80	18.80	16.90
Lowest Temperature in September (°C)	14.30	16.50	14.40
Lowest Temperature in October (°C)	11.10	13.30	11.20
Lowest Temperature in November (°C)	7.60	9.90	7.50
Lowest Temperature in December (°C)	4.90	7.10	4.70
